# Some Pitfalls of Practice

**Published:** 1897-01-02

**Authors:** 


### The Hospital

A Journal of

The Medical Sciences and Hospital Administration,

Vol. XXI.—No. 536.] [EARLY EDITION.] [Jan. 2, 1897.

### Some Pitfalls of Practice.

Object lessons, when thoroughly grasped by the mind, leave impressions behind which may be of service so long as the brain continues to retain its normal integrity. The whole profession has been sensitively, almost morbidly interested in the case of Stiven v. Welsford. It has been in every particular a "modern" case. Fifty years ago no such case as that of Stiven v. Welsford would have been possible; and for this reason, that, fifty years ago, diagnosis, as we understand it, was unknown. To-day diagnosis, we do not hesitate to say, is, in a scientific sense, the art of arts in medicine. Among ourselves, a practitioner, to a large extent, stands or falls by his capacity in diagnosis. The prevalent sensibilities of the profession are pervading the public mind. It is now beginning to be generally recognised and understood that diagnosis is the central fact upon which all rational treatment must depend.

The legal aspects of the case of Stiven *v.* Welsford undoubtedly have their interest; but, at the present stage of our progress as a profession, those are as nothing compared with the importance of the medical aspects. It is almost impossible for a medical man to hide from himself as he reads the details of this case how absorbingly important it is that a complete and accurate diagnosis should be arrived at in the earliest possible stage of every disease that comes before him. Not that we would make light of the difficulties which often exist, for in truth the beginnings of disease are often deceptive, and it is perhaps not unnatural that a medical man who is a friend as well as a doctor may sometimes find the wish leading the thought, and tempting him to incline to the less serious alternative. Nevertheless, the inexorable progress of a serious malady is sure before long to make evident its nature, and one cannot too strongly insist on the necessity of every medical man putting his wishes and hopes on one side, shutting his mind to the dictates of friendly sympathy, and forcing himself to take an outside, cold-blooded, and purely scientific view of the cases under his care.

The lasting impression which ought to be, and cannot but be, produced upon the minds of those who have followed this case in its developments from day to day, is that in every case to which the practitioner is called—not of possible appendicitis only, but of every kind—it ought to be held that, until a definite diagnosis is arrived at, no real progress has been really made. Of course the diagnosis may be "provisional"; it may be considered to be the outcome of much evidence or of little; it may be held as an "assured conviction" or as "highly probable," or only "slightly probable." But a diagnosis which shall reasonably satisfy the medical mind, and which shall be the outcome of the most impartial judgment the conscientious practitioner has the capacity to exercise, ought, we repeat, in every case to be made.

It may be said that this is only repeating what almost every medical lecturer insists upon at the opening of the session of his medical school. Most true, but what we wish to bring before the professional reader is the attitude of an attentive public towards the case of Stiven v. Welsford, and the demand which the public has gradually learnt to make upon what it considers to be the most scientific profession of modern times. The public has not cared in the least for the libellous or legal aspects of the case. What it has felt has been that, even in ailments which may seem but slight, the progress of the disease and the fate of the patient may depend on the capacity displayed by the attendant medical man to diagnose the case. To the public, a patient is not a case; he is a living man. Every thoughtful lay person perceives that he will be obliged to place himself entirely at the mercy of some doctor or other in case of his serious illness; and he reflects that a mistake in diagnosis may result in great disaster to himself.

In truth, modern medicine is a profession not without its terrors to those who follow it. So much may be demanded in the way of diagnosis and treatment at any moment even of men, who, in their ordinary daily work, are occupied with the most ordinary humdrum cases, and so certain is it that if they fail to rise to the occasion, half a dozen or half a hundred other men of the most competent experience and the most alert capacity may come in and expose their errors, that a doctor who has the least degree of imagination can hardly help feeling that he is walking on the thinnest of ice, on water which is fathoms deep, and that he may be hopelessly submerged and professionally drowned almost before he has time to turn round.

Between imperfect diagnosis and unsuccessful treatment there is all the difference in the world; and it is exactly here that the general community shows its capacity and justice in weighing up the merits or demerits of the medical practitioner. That treatment may fail; that an organ or the general condition of a patient's body may be such that treatment not only may but must fail, the public knows very well, just as it knows that there comes a time in the history of every house or castle when repairs, however skilfully designed, are no longer possible, and that the hour has struck when the structure must fall into ruins. And that being so, the public, as well as the profession, is very lenient towards want of success in treatment. But with diagnosis the case is entirely different. It is well known that the art of discovering the where and what of disease has attained great perfection, and the public expects that though the profession cannot always cure, it will, at least, thoroughly diagnose. If it does not diagnose, the public thinks it incompetent; and in this the public makes but small allowances for difficulties or complications. Many medical men deplore that cases like that of Stiven v. Welsford should be reported in the public newspapers. Not so we. Religion, politics, literature, law, and everything else which belongs to the scheme of the great world, must in the long run stand or fall by its loyal and capable service, or otherwise, for mankind at large. It ought to be, and is, exactly the same with physic; and we are glad to recognise the interest shown by the public in all things pertaining to the progressive science of medicine. Not the public only, but medicine itself will be stimulated and advantaged by criticism on the part of the non-medical world.

